# Micro-Environment Causes Reversible Changes in DNA Methylation and mRNA Expression Profiles in Patient-Derived Glioma Stem Cells

**DOI:** 10.1371/journal.pone.0094045

**Published:** 2014-04-11

**Authors:** Mehmet Baysan, Kevin Woolard, Serdar Bozdag, Gregory Riddick, Svetlana Kotliarova, Margaret C. Cam, Galina I. Belova, Susie Ahn, Wei Zhang, Hua Song, Jennifer Walling, Holly Stevenson, Paul Meltzer, Howard A. Fine

**Affiliations:** 1 New York University Cancer Institute, New York University Brain Tumor Center, New York University Langone Medical Center, New York, New York, United States of America; 2 Department of Pathology, Microbiology, and Immunology, University of California Davis, Davis, California, United States of America; 3 Department of Mathematics, Statistics, and Computer Science, Marquette University, Milwaukee, Wisconsin, United States of America; 4 Neuro-Oncology Branch, National Cancer Institute, National Institute of Neurological Disorders and Stroke, National Institutes of Health, Bethesda, Maryland, United States of America; 5 Genetics Branch, National Cancer Institute, National Institutes of Health, Bethesda, Maryland, United States of America; University Hospital of Navarra, Spain

## Abstract

*In vitro* and *in vivo* models are widely used in cancer research. Characterizing the similarities and differences between a patient's tumor and corresponding *in vitro* and *in vivo* models is important for understanding the potential clinical relevance of experimental data generated with these models. Towards this aim, we analyzed the genomic aberrations, DNA methylation and transcriptome profiles of five parental tumors and their matched *in vitro* isolated glioma stem cell (GSC) lines and xenografts generated from these same GSCs using high-resolution platforms. We observed that the methylation and transcriptome profiles of *in vitro* GSCs were significantly different from their corresponding xenografts, which were actually more similar to their original parental tumors. This points to the potentially critical role of the brain microenvironment in influencing methylation and transcriptional patterns of GSCs. Consistent with this possibility, *ex vivo* cultured GSCs isolated from xenografts showed a tendency to return to their initial *in vitro* states even after a short time in culture, supporting a rapid dynamic adaptation to the *in vitro* microenvironment. These results show that methylation and transcriptome profiles are highly dependent on the microenvironment and growth in orthotopic sites partially reverse the changes caused by *in vitro* culturing.

## Introduction

Glioblastoma Multiforme (GBM) is the most common and deadly primary brain tumor of the central nervous system. Developing experimental model systems that accurately recapitulate human tumor biology is critical for understanding the molecular pathogenesis of the disease as well as for developing and screening new therapeutics [1]–[7].

Completion of the human genome project and recent developments in high throughput molecular technologies have enabled the detailed genomic, epigenomic and transcriptome profiling of thousands of tumors in unprecedented detail. Specifically, a number of groups have used high resolution arrays to analyze the genomic aberrations [8]–[11], methylation alterations [12]–[16] and mRNA expression changes [17]–[20] found in human GBMs. Through the characterization of these genomic and epigenomic abnormalities comes not only an increased understanding of the biology of these tumors but also the potential of identifying new therapeutic targets. The validation that a given genetic aberration and/or a physiological process is a viable therapeutic target rests on the biological affects that result from perturbation of those targets in relevant *in vitro* and *in vivo* preclinical models. The closer those model systems are to the human disease, the greater the chance that those models will be predictive of clinically useful therapeutic agents. Others and we have previously shown that GBM-derived glioma initiating or GSCs more closely recapitulate the genotype and biology of primary human GBMs than do standard glioma cell lines [21]–[25]. Nevertheless, there are clearly some differences between parental tumors and derived cell lines at the DNA methylation and mRNA expression level [3]. Exactly how closely GSCs retain the genotype and epigenomic profile of their parental tumors after serial passage in *in vitro* and *in vivo*, however, is unknown.

In order to better understand the genomic and epigenomic changes that occur following the passage of GSCs *in vitro* and *in vivo*, we analyzed the genetic alterations, genomic methylation and the transcriptome profiles of several primary human GBM-derived GSCs *in vitro* and *in vivo*. We demonstrated that although the GSCs maintain similar genomic and epigenomic characteristics with their parental tumors, the *in vitro* and *in vivo* microenvironments exert profound, but partially reversible changes on the methylation and gene expression profiles of each GSC line. These data reveal that not only are the types of cells used important, but also how those cells are grown is pivotal for accurate modeling of the human disease.

## Materials and Methods

### Ethics Statement

Human brain tumor specimens are studied under the Protocol# 02C0140, “A Prospective National Study to Molecularly and Genetically Characterize Human Gliomas: The Glioma Molecular Diagnostic Initiative” approved by the Institutional Review Board of National Cancer Institute (FWA#00005897/IRB#00000001). Written informed consent from the donor or the next of kin was obtained for use of this sample in research and samples were maintained according to the NCI Institutional Review Board Regulations.

The animal research in this study was carried out in strict accordance with the recommendations in the Guide for the Care and Use of Laboratory Animals of the National Institutes of Health. The protocol (ASP NOB001) was approved by the National Cancer Institute Animal Care and Use Committee on the Ethics of Animal Experiments of NIH. All intracranial injection and procedures were performed under Ketamine-Xylazine combination anesthesia, and all efforts were made to minimize suffering.

### Patient Samples

Five different GBMs were used for this study and were obtained from surgical samples based on a prospective NCI intramural clinical tissue acquisition trial. These samples were provided as snap frozen sections. Pathological diagnosis of these samples was determined by the local institutional neuropathologist and centrally reviewed by two NIH neuropathologists according to WHO criteria [26].

### Glioma Stem Cells and Xenografts

Tumor cells were washed and enzymatically dissociated into single cells within three hours of surgical removal. NBE media, which consists of Neurobasal media (Invitrogen), N2 and B27 supplements (0.5× each; Invitrogen), human recombinant bFGF and EGF (50 ng/ml each; R&D Systems) was used to culture glioma stem cells (GSCs) (for details [21]).

For xenografts, GSCs were resuspended in 2 µl of HBSS and injected intracranially using stereotactic techniques into severe combined immunodeficiency mice (SCID/NCr mice with BALB/c background, female, aged 3 months, and weighing around 20–22 g, obtained from Charles River, Frederick, MD), according to animal study proposal approved by NCI Animal Use and Care Committee. Intracranial tumors were resected and resected tissues were used as *in vivo* samples. Portion of resected tissues were cultured again in the same media to develop the *ex vivo* samples. Xenograft samples and *ex vivo* cell cultures were controlled for mouse contamination by real time genomic PCR of either mouse- or human-specific GAPDH probe set (Applied Biosystems, Cat#4308313 or Cat#402869) and only samples that showed no detectable mouse tissue (or cell) contamination were used in subsequent analyses.

We predicted the G-CIMP status of each sample using our previously published prediction method [27]. We determined all samples as G-CIMP negative. Moreover we checked the IDH1 status of these samples with targeted sequencing and could not detect IDH1 R132 mutation.

### SNP Arrays and Data Set

QIAamp DNA kit (Qiagen) was used to prepare the genomic DNA from patient tumors, cultured GSCs and Xenografts. QIAamp DNA Blood Mini Kit (Qiagen) was used to prepare DNA from patient reference blood. Prepared DNA was hybridized onto arrays according to the manufacturer's recommendations (Affymetrix Human SNP array 6.0 and CytoScan HD array). After hybridization, the arrays were stained on the Affymetrix GeneChip fluidics station 450 and scanned at high resolution using the GeneChip Scanner 3000 7G.

Constructed CEL files were imported using Nexus (version 6.1). SNP-FASST2 segmentation was used for segmentation with 1000 Kbp as the maximum contiguous probe spacing and three as the minimum number of probes per segment. The significance threshold for segmentation was set at 5.0E-7. SNP-FASST2 Segmentation Algorithm is an extension of the FASST2 Segmentation Algorithm. FASST2 Segmentation Algorithm is a Hidden Markov Model (HMM) based approach that uses many states to cover more possibilities, such as mosaic events, and then make calls based on a second-level threshold. With the SNP-FASST2 algorithm, B-allele frequency probes are assigned to a range of possible states, which are used to make the final copy number and allelic event calls. The log ratio thresholds for single copy gain and single copy loss were set at 0.3 and −0.35, respectively. The log ratio thresholds for two or more copy gain and homozygous loss were set at 0.7 and −1.1, respectively.

### DNA Methylation Arrays and Data Set

DNA from cell pellets and fresh frozen tumor tissue was extracted with QIAmp DNA Micro Kit (Qiagen). One microgram of the DNA was bisulfite converted and processed on Human Methylation450 BeadChips (Illumina) using the Infinium HD Methylation Assay. Image data were extracted and analyzed using the GenomeStudio v2011.1 methylation module (Illumina). Methylation sites, which have detection p-value greater than 0.05 for any sample or have a missing value for any sample, were filtered out. We also filtered out methylation sites that reside on sex chromosomes to eliminate gender effect. 459,913 methylation sites remained after filtering. We ran batch controls for the batches that include five parental tumors-GSC pairs and did not detect a batch effect based on principle component analysis (PCA). We did not use batch controls for *in vitro*-*in vivo*-*ex vivo* triplicates based on this result and previous experience in our lab [27].

### mRNA Expression Arrays and Data Set

RNA was isolated and purified from cell pellets and fresh frozen tumor tissue using TRIZOL (Invitrogen) and PureLink RNA Mini Kit (Invitrogen). Affymetrix GeneChip Command Console (AGCC) was used to create the raw mRNA expression data (CEL files). We imported data to Partek Genomics Sofware (version 6.6, Partek Inc., St. Charles, MI) and normalized samples using RMA defaults (RMA background correction, quantile normalization, and median polish).

### Hierarchical Clustering (HC) and Principle Component Analysis (PCA)

We used Partek Genomics Software (version 6.6, Partek Inc., St. Charles, MI) to perform HC and PCA. Agglomerative (bottom-up) approach, complete linkage and Euclidean distance were used for all HCs. Expression data were standardized for each column (probe set) prior to performing the HC, although this was not done for the methylation data. PCAs were performed using correlation dispersion matrix and normalized eigenvector scaling.

## Results

### Comparison of genomic aberrations

**Figure 1:**
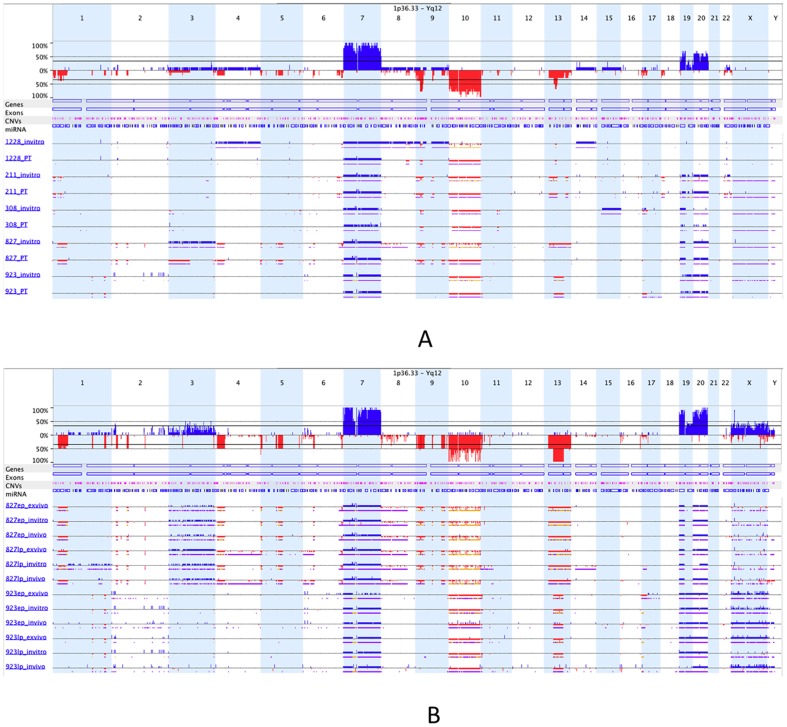
Copy number alterations for patient tumors and *in vitro*, *in vivo*, *ex vivo* GSC samples. (A) Comparison of five matched patient tumors and their corresponding *in vitro* GSCs. Each line represents a sample. Three or four digit code represents GSC id, PT represents patient tumor and invitro represents *in vitro* GSC. Blue is amplification, red is deletion, purple is loss of heterozygosity and orange is allelic imbalance. Examples of the many differences between PTs and matched *in vitro* GSCs are loss on chromosome 13 at GSC-827 and gain on chromosomes 4, 8, 9 and 14 at GSC-1228. (B) Comparison of *in vitro*-*in vivo*-*ex vivo* triplicates for early and late passages of two GSCs.

We used Affymetrix SNP6.0 and Cytoscan HD arrays to measure the genomic aberrations in our parental tumors (PTs), matched cultured GSC lines (*in vitro*) and GSC-generated xenografts (*in vivo*). To determine somatic changes, for each sample we subtracted background using blood DNA arrays from the same patient. First, we compared the genomic profiles for five matched patient tumor and *in vitro* GSCs (passage 10). We observed that there were some significant differences between genomic profiles of patient tumors and their corresponding GSCs (Figure 1A). These differences might be due to selective growth and/or loss of certain tumor sub-clones or acquisition of new aberrations in culture.

We next compared early and late passage (5 and 18, respectively) *in vitro* GSCs, the *in vivo* xenografts they formed and the GSCs cultured from those dissected xenografts (“*ex vivo*”). We observed that the genomic profiles in all matched samples were nearly identical (Figure 1B), consistent with our prior characterization of GSCs [21]. Based on these results, it appears that the initial selection of clonal subtypes in culture plays a major role in the copy number differences between patient tumors and GSCs, after which copy number alterations remain remarkably stable whether serially passaged *in vitro* or *in vivo*.

### Comparison of genomic DNA methylation

**Figure 2:**
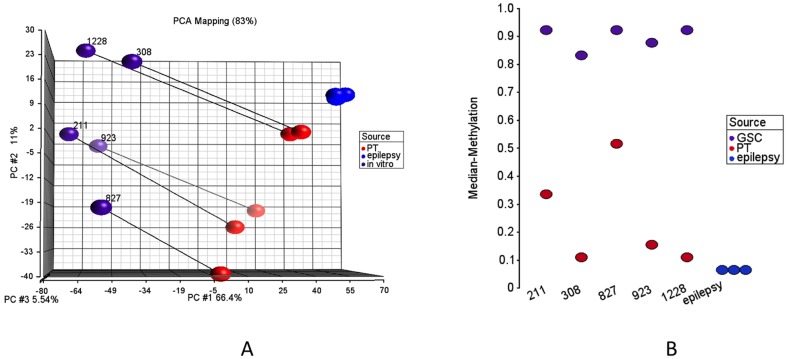
DNA methylation profiles for patient tumors and *in vitro* GSCs. (A) Principle Component Analyses (PCA) of 3847 methylation sites with standard deviation more than 0.35. PT represents patient tumor and in vitro represents the corresponding *in vitro* GSCs. Matched PT-*in vitro* pairs are connected with lines. (B) Median methylation values for each sample based on selected 3847 sites.

We used the Illumina Infinium 450K platform to assess methylation profiles of our samples [28], [29]. This platform measures methylation level for a methylation site as a continuous value between 0 and 1. It compares the methylated and unmethylated probe intensities on bisulfite treated DNA. For our analysis, we included five PT-*in vitro* pairs, four *in vitro-in vivo-ex vivo* triplicates and three non-tumor brain (epilepsy) samples. In first part of the analyses, we focused on potential changes between non-tumor brain tissues, patient tumors and *in vitro* GSCs. Figure 2A represents the summation of the methylation analyses through a PCA of 3847 methylation sites with standard deviation greater than 0.35. This figure demonstrates that all GSCs have significantly lower values in the first principal component (x-axis), which represents more than 60% of the variation. This suggests that there is a systematic change between PTs and *in vitro* GSCs. We also observe that all three non-tumor samples cluster together at the upper-right end of the plot completely away from the PTs and the GSCs. We checked the median methylation values to see whether methylation changes observed in *in vitro* GSCs is associated with an overall hypo- or hyper-methylation. We detected significant hyper-methylation in cultured cells, which was consistent for all five PT-*in vitro* pairs (Figure 2B). We also used HC to assess the similarity of samples and to validate our PCA results (Figure S1). In HC, 5 PTs with epilepsy samples and 5 *in vitro* GSCs clustered as two separate groups, which confirmed PCA results.

**Figure 3:**
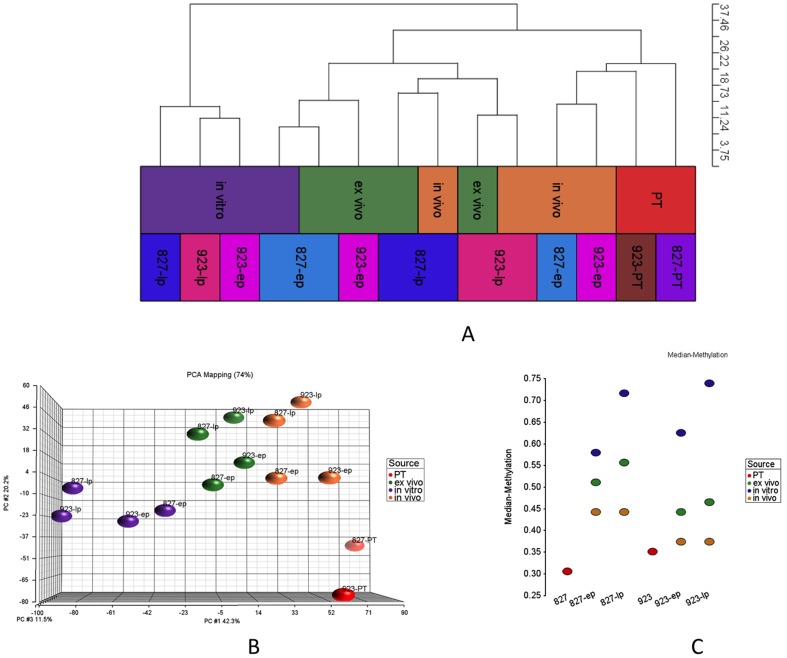
DNA methylation profiles of patient tumors and *in vitro*, *in vivo*, *ex vivo* GSCs for two cell lines. (A,B) HC and PCA for PT, *in vitro*, *in vivo* and *ex vivo* samples for early and late passages (ep, lp). 6825 sites with standard deviation greater than 0.15 are presented. These sites are not differentially methylated between 827 and 923 (Mann-Whitney p-value more than 0.5). (C) Median methylation values for each sample based on selected 6825 sites.

In the next part of our analyses, we focused on the differences between *in vitro* and *in vivo* models. For better assessment of the data, we removed the differentially methylated sites between two GSC models that we used in our analyses. Along this aim, we ran a Mann-Whitney test and retained 163,959 out of 459,913 methylation sites, which were not differentially methylated between GSCs 827 and 923 (p-value>0.5). Out of these sites, we present 6825 sites with standard deviation greater than 0.15 as HC and PCA (Figure 3A, 3B, S2).

We observed that *ex vivo* samples were clustered between *in* vitro and *in vivo* samples, which points to the partial recovery of micro-environmental associated changes in just a few passages (Figure 3A). Ideally, *ex vivo* samples were expected to cluster more closely to the *in vitro* samples, but perhaps since *ex vivo* samples spent less time in culture, they were not clustered tightly with *in vitro* samples (Figure 3A). GSCs derived from *ex vivo* samples, however, were much closer to *in vivo* samples, which might be due to less time in culture. We also observed that the changes seen with serial passage *in vitro* or through growth *in vivo* were highly similar between GSC lines derived from different patients (Figure 3B). Finally, we found that xenografts from early passage GSCs arewere much clustered together with PTs and xenografts from late passage GSCs were clustered with late passage *ex vivo* samples suggesting that the time in culture has an important effect on overall methylation profile. (Figure 3A, Figure 3C). The differences that were seen between xenografts and PTs (Figure 3B) could be the result of influence of mouse versus human brain microenvironments, the time that GSCs spent in culture before xenotransplantation, or the selection of specific xenograft-generating GSCs.

When we compared the median methylation values for all 827 and 923 samples, we observed that PTs and *in vivo* samples have lower median methylation levels than their corresponding *in vitro* and *ex vivo* samples (Figure 3C). We also observed that later passage *in vitro (ex vivo)* samples have much higher median methylation levels than earlier passage *in vitro (ex vivo)* samples. The differences between early and late passage *in vivo* samples, however, were much smaller than their corresponding *in vitro* GSCs, suggesting that the hyper-methylation seen with extended passaging is highly reversible.

### Comparison of Transcriptomes

**Figure 4:**
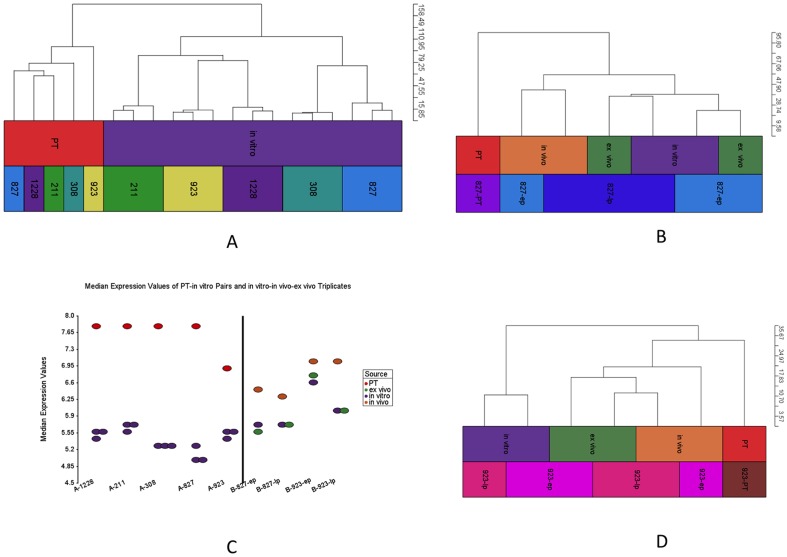
Trancriptome data for high variation (std. dev. >1.3) 1901 probe sets. (A) HC for matched PT-*in vitro* pairs for five GSC lines. (B) HC for PT and *in vitro-in vivo-ex vivo* triplicates for early and late passage 827 samples (C) Median expression values for all samples. (D) HC for PT and *in vitro-in vivo-ex vivo* triplicates for early and late passage 923 samples.

We compared seven matched PT-GSC pairs and four *in vitro*-*in vivo*-*ex vivo* matched samples at the transcriptome level using Affymetrix GeneChip Human Genome U133 Plus 2.0 arrays. We used U87 cells as replicates in different batches to control for changes due to technical artifacts. All U87 replicates clustered together in HC and PCA, which confirmed the quality of the results (Figure S3, S4). Next, we removed U87 replicates and imported remaining samples with into Partek with RMA normalization and filtered in high variation (st. dev. >1.3) 1901 probe sets (out of 54,678). The HC of these probe sets is represented in Figure 4A for five PT-*in vitro* GSC pairs (GSCs are passage 10 and three replicates were used), which demonstrates a clear separation between these two groups. Figure 4B and 4D represent HCs for PT, *in vitro*, *in vivo*, *ex vivo* samples for 827 and 923 samples, respectively. These HCs demonstrate a clear separation between PTs and *in vitro* GSCs, but a relative similarity between PTs and their corresponding xenografts. Thus, it appears that xenografts partially recover the gene expression profiles seen in the original PT. Finally, when we compared the median expression values, we observed that PTs and *in vivo* samples have higher expression levels compared to matched *in vitro* and *ex vivo* samples (Figure 4C).

When we looked at the methylation and expression data in total, we observed that PT and *in vitro* GSCs are very different in both data sets. By contrast, the PT samples were much more similar to *in vivo* xenografts than *in vitro* samples in both data sets.

### Detailed Analyses for Differentially Methylated and Expressed Genes

**Figure 5:**
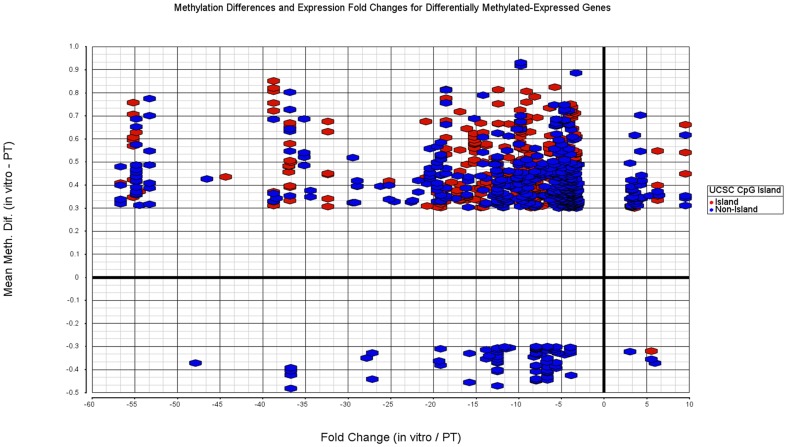
Fold changes (x-axis) and mean methylation differences (y-axis) between paired *in vitro*-PT pairs for both differentially expressed and differentially methylated genes. Differentially expressed genes determined with paired t-test. Genes with false discovery rate less than 0.05 (Benjamini-Hochberg) and absolute fold change greater than three are used. Differentially methylated sites determined with paired non-parametric Quade test and sites with false discovery rate less than 0.05 (Benjamini-Hochberg) and absolute methylation difference greater than 0.3 are used.

We next explored which genes demonstrated significant differences between five paired PT and *in vitro* GSCs both in DNA methylation and mRNA expression. We selected 22,264 (5408 genes) out of 459,913 methylation sites based on a non-parametric paired Quade test with Benjamini-Hochberg FDR<0.05 and mean methylation difference greater than 0.3. Similarly we ran a paired t-test and picked 645 differentially expressed genes with Benjamini-Hochberg FDR<0.05 and absolute fold change greater than three. The intersection between these gene sets demonstrated 238 differentially methylated and differentially expressed genes (Table S1). Figure 5 demonstrates these genes with mean methylation differences (y-axis) and fold changes (x-axis). Data points are colored red if it is a part of a CpG island and blue if it is not (all genes in Figure S5). We observed that the majority of differentially methylated and differentially expressed genes were hyper-methylated and down-regulated in *in vitro* GSCs. Furthermore, a significant proportion (p-value<0.0001 Chi-Squate test with Yates correction) of the hyper-methylated sites occured within CpG islands (365/802) while hypo-methylated sites rarely occur within CpG islands (2/78).

Next, we categorized methylation sites with respect to their locations on genes as TSS1500 (site location is between 1500 and 200 bases upstream of transcription start site (TSS)), TSS200 (site location is within 200 bases upstream of TSS), 5′ UTR, 1^st^ Exon, Body (except 1^st^ exon) and 3′ UTR. Out of the 64,671 sites in TSS1500, 3536 (5.47%) are differentially methylated. Similarly; 4.74%, 4.72%, 4.15%, 4.02% and 3.82% of the sites in the 1^st^ Exon, 5′ UTR, Body, TSS200 and 3′ UTR are differentially methylated. This shows that sites in the promoter, but not in the immediate vicinity of TSS, were preferentially targeted for methylation changes *in vitro*. Next, for methylation sites associated with genes, we measured which portion of differentially methylated genes is also differentially expressed. Out of the differentially methylated sites within each region, 7.18%, 7.00%, 6.69%, 5.32%, 4.73% and 3.63% are also differentially expressed in 5′ UTR, 1^st^ Exon, TSS200, TSS1500, Body, and 3′ UTR, respectively, which shows stronger methylation-expression interaction near TSS.

Since CpG islands are very important in terms of DNA methylation, we categorized methylated sites according to their location with respect to CpG islands. We defined four categories as Island, Shore (0–2000 base pairs to Island), Shelf (2000–4000 base pairs to Island) and Rest and assigned each site to one of these categories. We observed that 7.03%, 5.60%, 3.27% and 2.22% of the sites are differentially methylated in Island, Shore, Rest and Shelf categories, respectively. This shows that sites located in or close to CpG Islands are more sensitive to *in vitro* changes. Then, we measured which percent of the differentially methylated genes are differentially expressed within each region. We observed that 5.78%, 3.33%, 1.34% and 0.40% of the differentially methylated sites within Island, Shore, Rest and Shelf categories are also differentially expressed, respectively. This suggests that methylation-expression regulation is stronger in CpG islands.

A subset of GBM samples are categorized as G-CIMP due to the hyper-methylation profile they display [12]. This hyper-methylation profile is tightly associated with a mutation in IDH1 [14]. We, therefore, checked whether the methylation sites, which show differences between G-CIMP positive, and G-CIMP negative GBMs, were also differentially methylated between parental tumors and *in vitro* samples. To this point, we analyzed 368 TCGA samples from our previous study [27]. There were 21,587 methylation sites shared between that data set and the data set we used for this study. Among these, 936 and 854 sites were differentially methylated between G-CIMP positive and negative samples (Mann-Whitney, Benjamini-Hochberg FDR<0.05 and mean methylation difference >0.3) and between parental tumors and *in vitro* samples (Quade, Benjamini-Hochberg FDR<0.05 and mean methylation difference >0.3), respectively. 271 sites were shared between two comparisons. When we ran a Chi-Square test with Yates correction on these numbers, we observed that differentially methylated sites in the two comparisons were associated (p-value<0.001). This suggests that certain sites have a tendency for methylation changes regardless of the specific genetic or environmental context.

Finally, we analyzed the *in vitro* differentially methylated and differentially expressed 238 genes with fold changes using Ingenuity Pathway Analyses software for functional enrichment. We observed development and cancer related categories such as “Hematological System Development”, “Cancer”, “Cellular Development” and “Cell Death and Survival” as the most enriched categories (Figure S6).

### Genes potentially involved in GSC DNA hyper-methylation in vitro

Serial cell passage *in vitro* is known to change the methylation profiles of cultured cells [30]–[32]. We confirmed this finding in this study as we observed hyper-methylation in *in vitro* samples compared to PTs and progressive hyper-methylation with serial passage *in vitro*. To identify the potential genes that might be related to this *in vitro* hyper-methylation phenotype, we compared the expression levels of genes encoding proteins with “methylation” functions based on Gene Ontology (GO) categories [33]. We identified 220 genes (out of 21,121 genes in our data set) within this category. In a paired t-test comparing five different matched PTs and *in vitro* GSCs, we identified 12 differentially expressed genes with FDR less than 0.03. These genes are ATF7IP, BCDIN3D, DNMT1, ELP2, GATAD2A, GSPT1, MTA2, N6AMT1, NTMT1, PRMT5, TPMT and WDR5. In a similar fashion, we ran paired t-tests between four matched *in vitro*-*in vivo* and *in vivo-ex vivo* pairs. We obtained 89 and 77 differentially expressed methylation-related genes, respectively with p-value less than 0.03 (FDR<0.23). ATF7IP, ELP2, NTMT1, PRMT5 and TPMT are the five genes that were present in all three comparisons. Among these genes, PRMT5 has been recently reported to cause DNA methylation changes [34]. PRMT5 mediates methylation of histone H4R3, which recruits DNMT3A resulting in DNA methylation and repression of gene expression. Consistent with this, we observed clear differences between PT-*in vitro* and *in vitro*-*in vivo* pairs for PRMT5 expression (Figure S7). These results suggest that up-regulation of PRMT5 expression *in vitro* may partially contribute to the genomic hyper-methylation seen in GSCs with serial passage *in vitro*.

## Discussion

In this study, we characterized the genomic aberration, DNA methylation and mRNA expression profiles of parental GBMs with their matched *in vitro* and *in vivo* GSCs that they generate. Although earlier studies have addressed some aspects of the differences between parental tumor and their matched tumor cell lines, our study is one of the first to use high-resolution arrays to profile multiple dimensions of the genomic, methylation and transcription machinery in detail. Our results show that DNA methylation and mRNA transcription undergo significant and reproducible transformation from *in vitro* to *in vivo* growth conditions and then back again. These changes were seen when comparing PTs to their corresponding GSCs *in vitro*, and were partially reversed when those same GSCs formed xenograft tumors in the brains of immunodeficient mice. When we cultured GSCs from those same xenograft tumors, we observed a quick reversion back to the original *in vitro* GSC-related expression profiles within a few passages, with a much slower and less complete recovery of their methylation profiles. In summary, our observations demonstrate that GSCs quickly adapt to their microenvironment by changing their transcriptome and epigenome.

We observed a reduction in the overall mRNA expression levels in *in vitro* GSCs compared to PTs and *in vivo* GSCs. The reasons for this are unclear but are likely multifactorial. One possibility might be due to down-regulation *in vitro* of a number of pathways, which normally responsive to the diverse extra-cellular-signaling environment found *in vivo*. Another possibility rests on the observation made by others and us that GSC mediated xenograft formation requires high levels of c-myc *in vivo* in contrast to *in vitro* GSC proliferation that requires much lower levels [35]. Given the recent demonstration of c-myc as an enhancer of overall transcriptional levels throughout the genome [27], lower levels of c-myc activity in *in vitro* GSCs may contribute to the overall lower mRNA transcriptional levels in *in vitro*. Finally, the progressive increase in genomic methylation that others and we observed with serial passage of our GSCs may be representative of and/or contribute to an overall increase in the heterochromatin content of the genome of *in vitro* passaged GSCs thereby resulting in a reduction in mRNA expression.

Our data demonstrate the adaptation of glioma cells to their microenvironment but it does not explain the mechanism of adaptation. One possibility is that the selective pressures of the given microenvironment (*in vitro* or *in vivo*) can select for an adaptive phenotype and genotype most advantageous for those pressures. Another, non-mutually exclusive possibility, is based on the fact that GBMs and GSC lines are made up of highly heterogeneous clones of tumor cells and genomic profiles obtained from tumors represent only an averaged result for potentially innumerable clones. Individual clones may be selected for by *in vitro* and *in vivo* selective pressure causing a dominant clone(s), with its corresponding genotype and phenotype, to dominate. The existing data do not allow us to definitively discriminate between these two possibilities although it is likely that both mechanisms play a role in explaining the changing profiles seen between the different growth conditions. Further studies to examine the clonal distribution will be required to explain the mechanistic basis for how GSCs adapt to their microenvironment.

In our analyses, we observed that *in vivo* xenografts were clustered between PTs and *in vitro*/*ex vivo* samples in HCs on DNA methylation (Figure 3A) and mRNA expression (Figure 4B, 4D) datasets. We also detected significant and consistent differences between PTs and *in vivo* xenografts. One potential reason for this could be contamination of normal human or mouse brain tissue. To exclude this potential problem, we applied strict contamination controls as measured by qRT-PCR for human and mouse specific probes, and only used samples with no detectable (or negligible) contamination. Thus, it is again likely that the differences seen between the expression and methylation profiles of the PTs and their corresponding xenografts are either due to initial elimination of certain PT cells in culture, selection of specific clones most adapted to growth in the murine central nervous system microenvironment and/or the biological adaptation of the tumor to the selective pressures of that unique microenvironment.

In our previous study, we have demonstrated that NBE grown GSCs are better representative of human GBMs than matched cells gown in serum or standard glioma cell lines [21]. In this study, we further define the limits of the GSC model for reproducing the biology of the human disease. The mechanisms driving these differences are beyond the scope of this report and additional studies examining the potential role of individual clones or cellular subpopulations *in vitro* cultures and *in vivo* will be necessary. Nevertheless, through the continued refinement of models such as these and through our increased understanding of the strengths and limitations of such models, will come a better tool for understanding GBM biology and more accurate predictive screening of novel therapeutic strategies.

## Supporting Information

Figure S1 **Hierarchical Clustering of methylation sites for non-tumor, patient tumor and in vitro, in vivo and ex vivo samples.** 3847 sites with standard deviation greater than 0.35 are presented. First column represents the type of sample and second column represents the GSC code. Each cell in the heat map is colored by the methylation rate; bright blue is 0% and bright red is 100% methylation.
(DOCX)

Figure S2 **Hierarchical Clustering for non-tumor, patient tumor, in vitro, in vivo and ex vivo samples.** 6825 sites with standard deviation greater than 0.15 are presented. These sites are not differentially methylated between 827 and 923 (Mann-Whitney p-value more than 0.5). First column represents the type of sample and second column represents the GSC code. Each cell in the heat map is colored by the methylation rate; bright blue is 0% and bright red is 100% methylation.
(DOCX)

Figure S3 **PCA for PT, in vitro, in vivo, ex vivo and U87 mRNA profiles.** Samples are imported with RMA and 3002/54678 probe sets with standard deviation greater than 1.3 are presented.
(DOCX)

Figure S4 **Hierarchical clustering for PT, in vitro, in vivo, ex vivo and U87 mRNA profiles.** Samples are imported with RMA and 3002/54678 probe sets with standard deviation greater than 1.3 are presented.
(DOCX)

Figure S5 **Fold changes (x-axis) and mean methylation differences (y-axis) between paired in vitro-PT pairs for both differentially expressed and differentially methylated genes.** Differentially expressed genes determined with paired t-test. Genes with false discovery rate less than 0.05 (Benjamini-Hochberg) and absolute fold change greater than three are used. Differentially methylated sites determined with paired non-parametric Quade test and sites with false discovery rate less than 0.05 (Benjamini-Hochberg) and absolute methylation difference greater than 0.3 are colored red and blue others are colored gray.
(DOCX)

Figure S6 **In vitro differentially methylated and differentially expressed 238 genes with fold changes are uploaded Ingenuity Pathway Analyses software for functional enrichment.** These are the enriched most categories; blue sub-categories are inhibited and orange ones are activated in vitro.
(DOCX)

Figure S7 **PRMT5 expression for matched PT, in vitro, in vivo and ex vivo samples.** Each column represents a matched samples and y-axis is the PRMT5 expression value.
(DOCX)

Table S1 **List of differentially methylated and differentially expressed genes.**
(TXT)

## References

[1] Chen J, McKay RM, Parada LF (2012) Malignant glioma: lessons from genomics, mouse models, and stem cells. Cell 149: 36–47. 22464322 10.1016/j.cell.2012.03.009 PMC3719882

[2] Jones TS, Holland EC (2011) Animal models for glioma drug discovery. Expert Opinion on Drug Discovery 6: 1271–1283. 22647066 10.1517/17460441.2011.632628

[3] Bhat KP, Balasubramaniyan V, Vaillant B, Ezhilarasan R, Hummelink K, et al (2013) Mesenchymal differentiation mediated by NF-κB promotes radiation resistance in glioblastoma. Cancer cell 24: 331–346. 23993863 10.1016/j.ccr.2013.08.001 PMC3817560

[4] Soda Y, Marumoto T, Friedmann-Morvinski D, Soda M, Liu F, et al (2011) Transdifferentiation of glioblastoma cells into vascular endothelial cells. Proceedings of the National Academy of Sciences 108: 4274–4280. 10.1073/pnas.1016030108 PMC3060261 21262804

[5] Singh D, Chan JM, Zoppoli P, Niola F, Sullivan R, et al (2012) Transforming fusions of FGFR and TACC genes in human glioblastoma. Science 337: 1231–1235. 22837387 10.1126/science.1220834 PMC3677224

[6] Denysenko T, Gennero L, Roos MA, Melcarne A, Juenemann C, et al (2010) Glioblastoma cancer stem cells: heterogeneity, microenvironment and related therapeutic strategies. Cell biochemistry and function 28: 343–351. 20535838 10.1002/cbf.1666

[7] Sung KE, Yang N, Pehlke C, Keely PJ, Eliceiri KW, et al (2011) Transition to invasion in breast cancer: a microfluidic in vitro model enables examination of spatial and temporal effects. Integrative Biology 3: 439–450. 21135965 10.1039/c0ib00063a PMC3094750

[8] Beroukhim R, Getz G, Nghiemphu L, Barretina J, Hsueh T, et al (2007) Assessing the significance of chromosomal aberrations in cancer: methodology and application to glioma. Proceedings of the National Academy of Sciences 104: 20007–20012. 10.1073/pnas.0710052104 PMC2148413 18077431

[9] Brennan CW, Verhaak RG, McKenna A, Campos B, Noushmehr H, et al (2013) The somatic genomic landscape of glioblastoma. Cell 155: 462–477. 24120142 10.1016/j.cell.2013.09.034 PMC3910500

[10] Frattini V, Trifonov V, Chan JM, Castano A, Lia M, et al (2013) The integrated landscape of driver genomic alterations in glioblastoma. Nature genetics 45: 1141–1149. 23917401 10.1038/ng.2734 PMC3799953

[11] Szerlip NJ, Pedraza A, Chakravarty D, Azim M, McGuire J, et al (2012) Intratumoral heterogeneity of receptor tyrosine kinases EGFR and PDGFRA amplification in glioblastoma defines subpopulations with distinct growth factor response. Proceedings of the National Academy of Sciences 109: 3041–3046. 10.1073/pnas.1114033109 PMC3286976 22323597

[12] Noushmehr H, Weisenberger DJ, Diefes K, Phillips HS, Pujara K, et al (2010) Identification of a CpG Island Methylator Phenotype that Defines a Distinct Subgroup of Glioma. Cancer Cell 17: 510–522. 20399149 10.1016/j.ccr.2010.03.017 PMC2872684

[13] Duncan CG, Barwick BG, Jin G, Rago C, Kapoor-Vazirani P, et al (2012) A heterozygous IDH1R132H/WT mutation induces genome-wide alterations in DNA methylation. Genome research 22: 2339–2355. 22899282 10.1101/gr.132738.111 PMC3514664

[14] Turcan S, Rohle D, Goenka A, Walsh LA, Fang F, et al (2012) IDH1 mutation is sufficient to establish the glioma hypermethylator phenotype. Nature 10.1038/nature10866 PMC3351699 22343889

[15] Bozdag S, Li A, Riddick G, Kotliarov Y, Baysan M, et al (2013) Age-specific signatures of glioblastoma at the genomic, genetic, and epigenetic levels. PloS one 8: e62982. 23658659 10.1371/journal.pone.0062982 PMC3639162

[16] Ene CI, Edwards L, Riddick G, Baysan M, Woolard K, et al (2012) Histone demethylase Jumonji D3 (JMJD3) as a tumor suppressor by regulating p53 protein nuclear stabilization. PloS one 7: e51407. 23236496 10.1371/journal.pone.0051407 PMC3517524

[17] Li A, Walling J, Ahn S, Kotliarov Y, Su Q, et al (2009) Unsupervised analysis of transcriptomic profiles reveals six glioma subtypes. Cancer Research 69: 2091–2099. 19244127 10.1158/0008-5472.CAN-08-2100 PMC2845963

[18] Gonda DD, Cheung VJ, Muller KA, Goyal A, Carter BS, et al. (2013) The Cancer Genome Atlas expression profiles of low-grade gliomas. 10.3171/2012.12.focus12351 24812719

[19] Verhaak RGW, Hoadley KA, Purdom E, Wang V, Qi Y, et al (2010) Integrated genomic analysis identifies clinically relevant subtypes of glioblastoma characterized by abnormalities in PDGFRA, IDH1, EGFR, and NF1. Cancer cell 17: 98–110. 20129251 10.1016/j.ccr.2009.12.020 PMC2818769

[20] Sumazin P, Yang X, Chiu H-S, Chung W-J, Iyer A, et al (2011) An extensive microRNA-mediated network of RNA-RNA interactions regulates established oncogenic pathways in glioblastoma. Cell 147: 370–381. 22000015 10.1016/j.cell.2011.09.041 PMC3214599

[21] Lee J, Kotliarova S, Kotliarov Y, Li A, Su Q, et al (2006) Tumor stem cells derived from glioblastomas cultured in bFGF and EGF more closely mirror the phenotype and genotype of primary tumors than do serum-cultured cell lines. Cancer Cell 9: 391–403. 16697959 10.1016/j.ccr.2006.03.030

[22] Chen J, Li Y, Yu T-S, McKay RM, Burns DK, et al (2012) A restricted cell population propagates glioblastoma growth after chemotherapy. Nature 488: 522–526. 22854781 10.1038/nature11287 PMC3427400

[23] Ricci-Vitiani L, Pallini R, Biffoni M, Todaro M, Invernici G, et al (2010) Tumour vascularization via endothelial differentiation of glioblastoma stem-like cells. Nature 468: 824–828. 21102434 10.1038/nature09557

[24] Bonavia R, Mukasa A, Narita Y, Sah DW, Vandenberg S, et al (2010) Tumor heterogeneity is an active process maintained by a mutant EGFR-induced cytokine circuit in glioblastoma. Genes & development 24: 1731–1745. 20713517 10.1101/gad.1890510 PMC2922502

[25] Persano L, Rampazzo E, Basso G, Viola G (2013) Glioblastoma cancer stem cells: role of the microenvironment and therapeutic targeting. Biochemical pharmacology 85: 612–622. 23063412 10.1016/j.bcp.2012.10.001

[26] Kleihues P, Louis DN, Scheithauer BW, Rorke LB, Reifenberger G, et al (2002) The WHO classification of tumors of the nervous system. Journal of Neuropathology & Experimental Neurology 61: 215–225. 11895036 10.1093/jnen/61.3.215

[27] Baysan M, Bozdag S, Cam MC, Kotliarova S, Ahn S, et al (2012) G-Cimp Status Prediction Of Glioblastoma Samples Using mRNA Expression Data. PloS ONE 7: e47839. 23139755 10.1371/journal.pone.0047839 PMC3490960

[28] Bibikova M, Barnes B, Tsan C, Ho V, Klotzle B, et al (2011) High density DNA methylation array with single CpG site resolution. Genomics 98: 288–295. 21839163 10.1016/j.ygeno.2011.07.007

[29] Dedeurwaerder S, Defrance M, Calonne E, Denis H, Sotiriou C, et al (2011) Evaluation of the Infinium Methylation 450K technology. Epigenomics 3: 771–784. 22126295 10.2217/epi.11.105

[30] Nazor KL, Altun G, Lynch C, Tran H, Harness JV, et al (2012) Recurrent variations in DNA methylation in human pluripotent stem cells and their differentiated derivatives. Cell Stem Cell 10: 620–634. 22560082 10.1016/j.stem.2012.02.013 PMC3348513

[31] Bork S, Pfister S, Witt H, Horn P, Korn B, et al (2010) DNA methylation pattern changes upon long-term culture and aging of human mesenchymal stromal cells. Aging Cell 9: 54–63. 19895632 10.1111/j.1474-9726.2009.00535.x PMC2814091

[32] Meissner A, Mikkelsen TS, Gu H, Wernig M, Hanna J, et al (2008) Genome-scale DNA methylation maps of pluripotent and differentiated cells. Nature 454: 766–770. 18600261 10.1038/nature07107 PMC2896277

[33] Ashburner M, Ball CA, Blake JA, Botstein D, Butler H, et al (2000) Gene Ontology: tool for the unification of biology. Nature Genetics 25: 25. 10802651 10.1038/75556 PMC3037419

[34] Zhao Q, Rank G, Tan YT, Li H, Moritz RL, et al (2009) PRMT5-mediated methylation of histone H4R3 recruits DNMT3A, coupling histone and DNA methylation in gene silencing. Nature Structural & Molecular Biology 16: 304–311. 10.1038/nsmb.1568 PMC5120857 19234465

[35] Kim HS, Woolard K, Lai C, Bauer PO, Maric D, et al (2012) Gliomagenesis arising from Pten-and Ink4a/Arf-deficient neural progenitor cells is mediated by the p53-Fbxw7/Cdc4 pathway, which controls c-Myc. Cancer Research 72: 6065–6075. 22986743 10.1158/0008-5472.CAN-12-2594

