# Supplementary material for: Micro-Environment Causes Reversible Changes in DNA Methylation and mRNA Expression Profiles in Patient-Derived Glioma Stem Cells
Source: PLoS One. 2014 Apr 11;9(4):e94045. doi: 10.1371/journal.pone.0094045 (PMC3984100; doi:10.1371/journal.pone.0094045)
Supplement: Figure S1 — Hierarchical Clustering of methylation sites for non-tumor, patient tumor and in vitro, in vivo and ex vivo samples. 3847 sites with standard deviation greater than 0.35 are presented. First column represents the type of sample and second column represents the GSC code. Each cell in the heat map is colored by the methylation rate; bright blue is 0% and bright red is 100% methylation. (DOCX) [file pone.0094045.s001.docx]

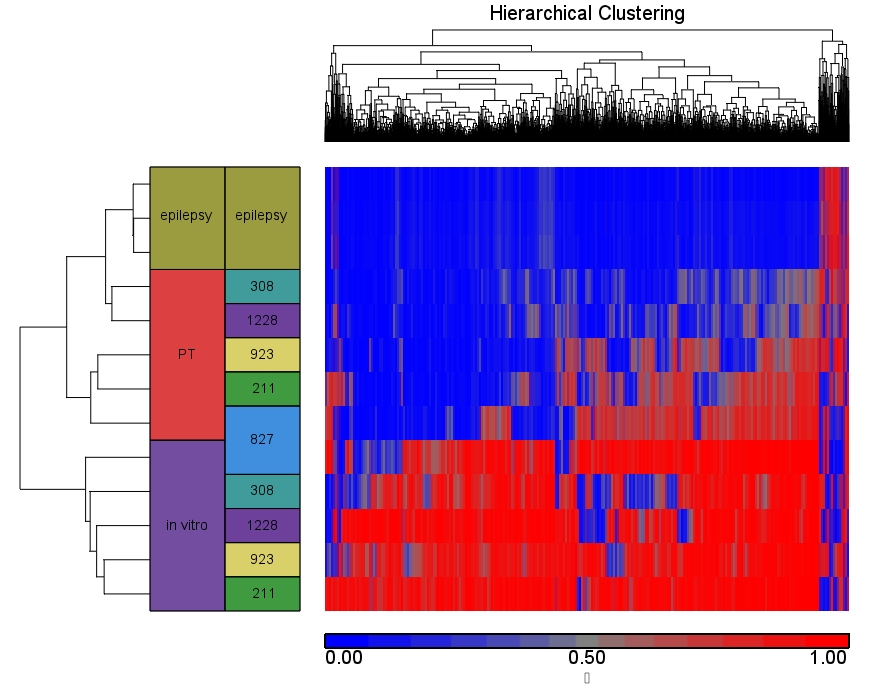


Figure S1: Hierarchical Clustering of methylation sites for non-tumor, patient tumor and *in vitro*, *in vivo* and *ex vivo* samples. 3847 sites with standard deviation greater than 0.35 are presented. First column represents the type of sample and second column represents the GSC code. Each cell in the heat map is colored by the methylation rate; bright blue is 0% and bright red is 100% methylation.
