# Supplementary material for: Micro-Environment Causes Reversible Changes in DNA Methylation and mRNA Expression Profiles in Patient-Derived Glioma Stem Cells
Source: PLoS One. 2014 Apr 11;9(4):e94045. doi: 10.1371/journal.pone.0094045 (PMC3984100; doi:10.1371/journal.pone.0094045)
Supplement: Figure S2 — Hierarchical Clustering for non-tumor, patient tumor, in vitro, in vivo and ex vivo samples. 6825 sites with standard deviation greater than 0.15 are presented. These sites are not differentially methylated between 827 and 923 (Mann-Whitney p-value more than 0.5). First column represents the type of sample and second column represents the GSC code. Each cell in the heat map is colored by the methylation rate; bright blue is 0% and bright red is 100% methylation. (DOCX) [file pone.0094045.s002.docx]

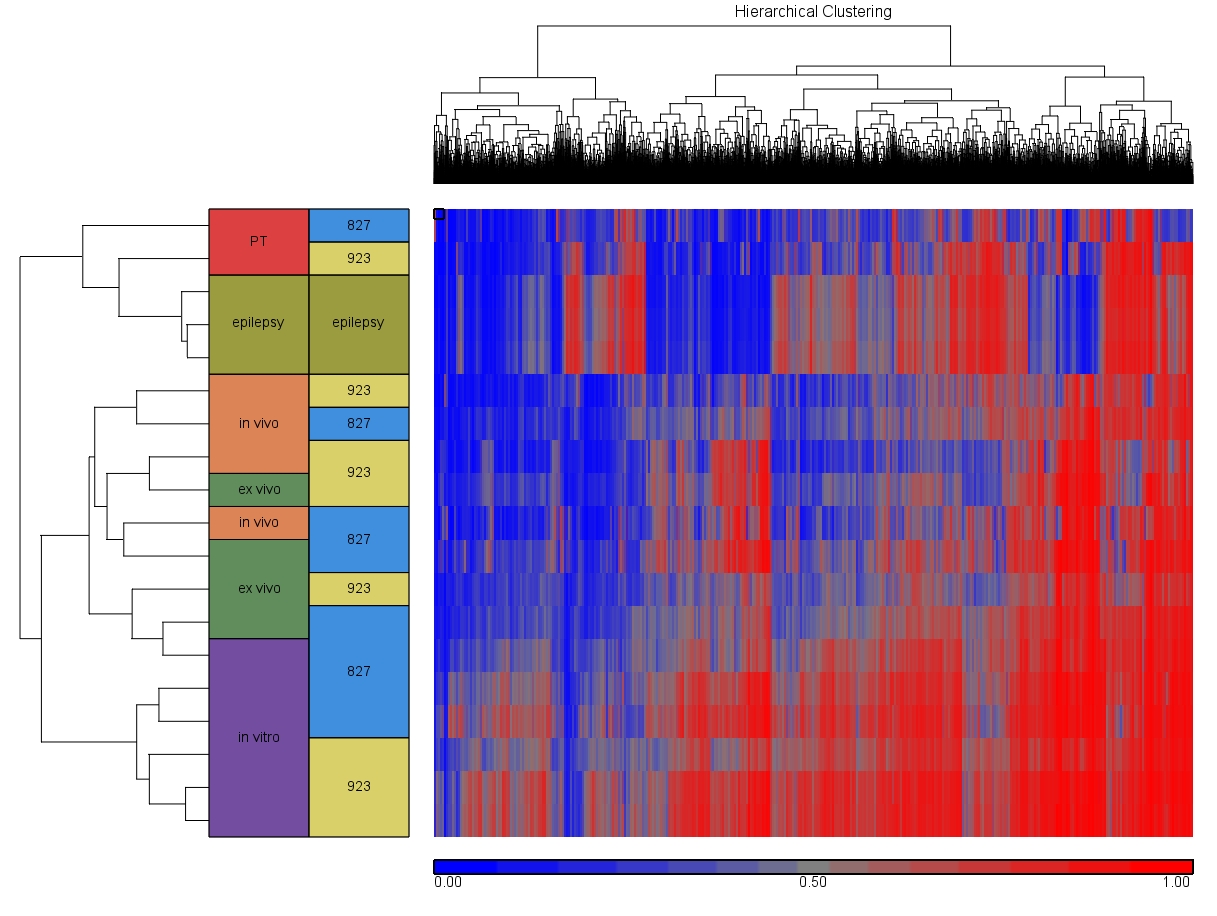


Figure S2: Hierarchical Clustering for non-tumor, patient tumor, *in vitro*, *in vivo* and *ex vivo* samples. 6825 sites with standard deviation greater than 0.15 are presented. These sites are not differentially methylated between 827 and 923 (Mann-Whitney p-value more than 0.5). First column represents the type of sample and second column represents the GSC code. Each cell in the heat map is colored by the methylation rate; bright blue is 0% and bright red is 100% methylation.
