# Supplementary material for: Micro-Environment Causes Reversible Changes in DNA Methylation and mRNA Expression Profiles in Patient-Derived Glioma Stem Cells
Source: PLoS One. 2014 Apr 11;9(4):e94045. doi: 10.1371/journal.pone.0094045 (PMC3984100; doi:10.1371/journal.pone.0094045)
Supplement: Figure S4 — Hierarchical clustering for PT, in vitro, in vivo, ex vivo and U87 mRNA profiles. Samples are imported with RMA and 3002/54678 probe sets with standard deviation greater than 1.3 are presented. (DOCX) [file pone.0094045.s004.docx]

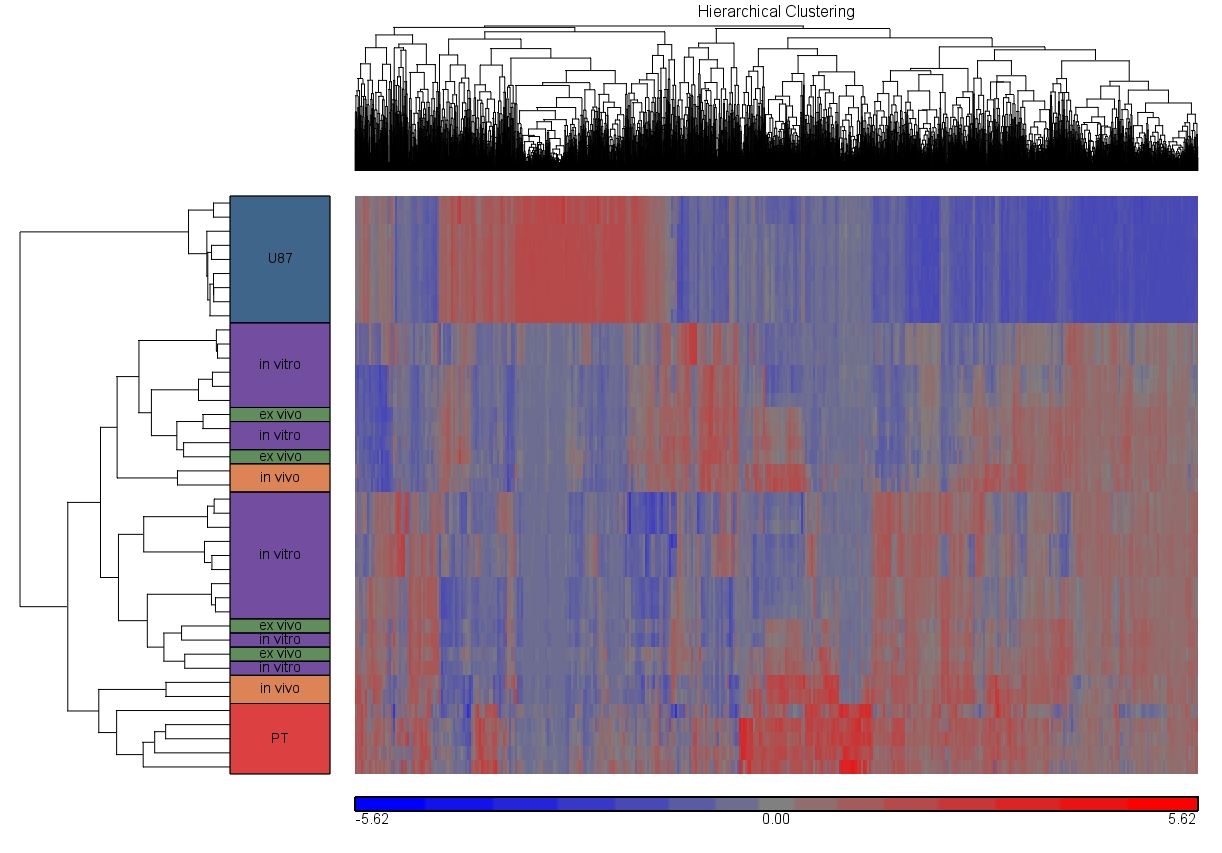


Figure S4: Hierarchical clustering for PT, *in vitro, in vivo,* *ex vivo* and U87 mRNA profiles. Samples are imported with RMA and 3002/54678 probe sets with standard deviation greater than 1.3 are presented.
