# Supplementary material for: Micro-Environment Causes Reversible Changes in DNA Methylation and mRNA Expression Profiles in Patient-Derived Glioma Stem Cells
Source: PLoS One. 2014 Apr 11;9(4):e94045. doi: 10.1371/journal.pone.0094045 (PMC3984100; doi:10.1371/journal.pone.0094045)
Supplement: Figure S5 — Fold changes (x-axis) and mean methylation differences (y-axis) between paired in vitro-PT pairs for both differentially expressed and differentially methylated genes. Differentially expressed genes determined with paired t-test. Genes with false discovery rate less than 0.05 (Benjamini-Hochberg) and absolute fold change greater than three are used. Differentially methylated sites determined with paired non-parametric Quade test and sites with false discovery rate less than 0.05 (Benjamini-Hochberg) and absolute methylation difference greater than 0.3 are colored red and blue others are colored gray. (DOCX) [file pone.0094045.s005.docx]

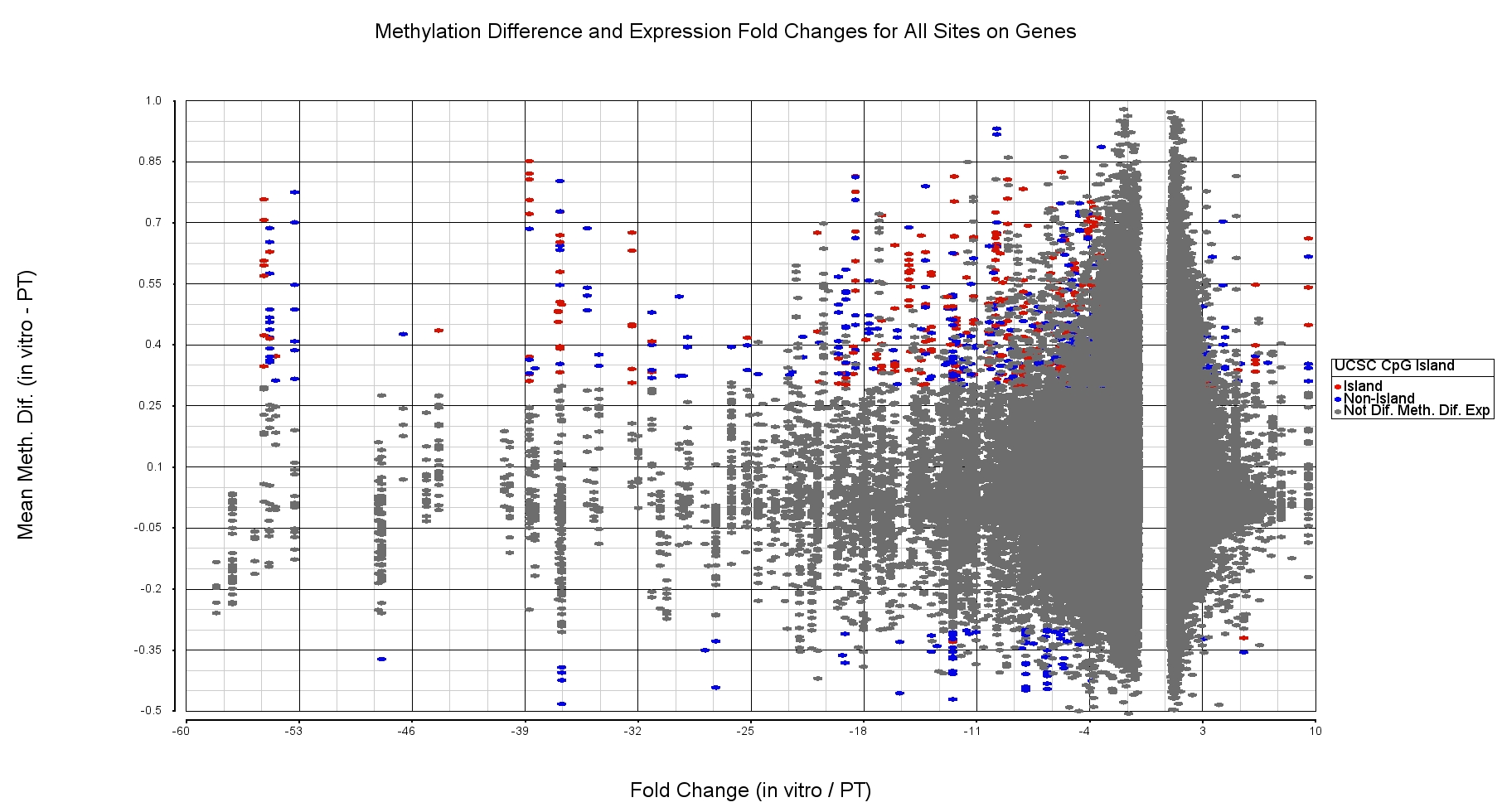


Figure S5: Fold changes (x-axis) and mean methylation differences (y-axis) between paired *in vitro*-PT pairs for both differentially expressed and differentially methylated genes. Differentially expressed genes determined with paired t-test. Genes with false discovery rate less than 0.05 (Benjamini-Hochberg) and absolute fold change greater than three are used. Differentially methylated sites determined with paired non-parametric Quade test and sites with false discovery rate less than 0.05 (Benjamini-Hochberg) and absolute methylation difference greater than 0.3 are colored red and blue others are colored gray.
