# Supplementary material for: Micro-Environment Causes Reversible Changes in DNA Methylation and mRNA Expression Profiles in Patient-Derived Glioma Stem Cells
Source: PLoS One. 2014 Apr 11;9(4):e94045. doi: 10.1371/journal.pone.0094045 (PMC3984100; doi:10.1371/journal.pone.0094045)
Supplement: Figure S6 — In vitro differentially methylated and differentially expressed 238 genes with fold changes are uploaded Ingenuity Pathway Analyses software for functional enrichment. These are the enriched most categories; blue sub-categories are inhibited and orange ones are activated in vitro. (DOCX) [file pone.0094045.s006.docx]

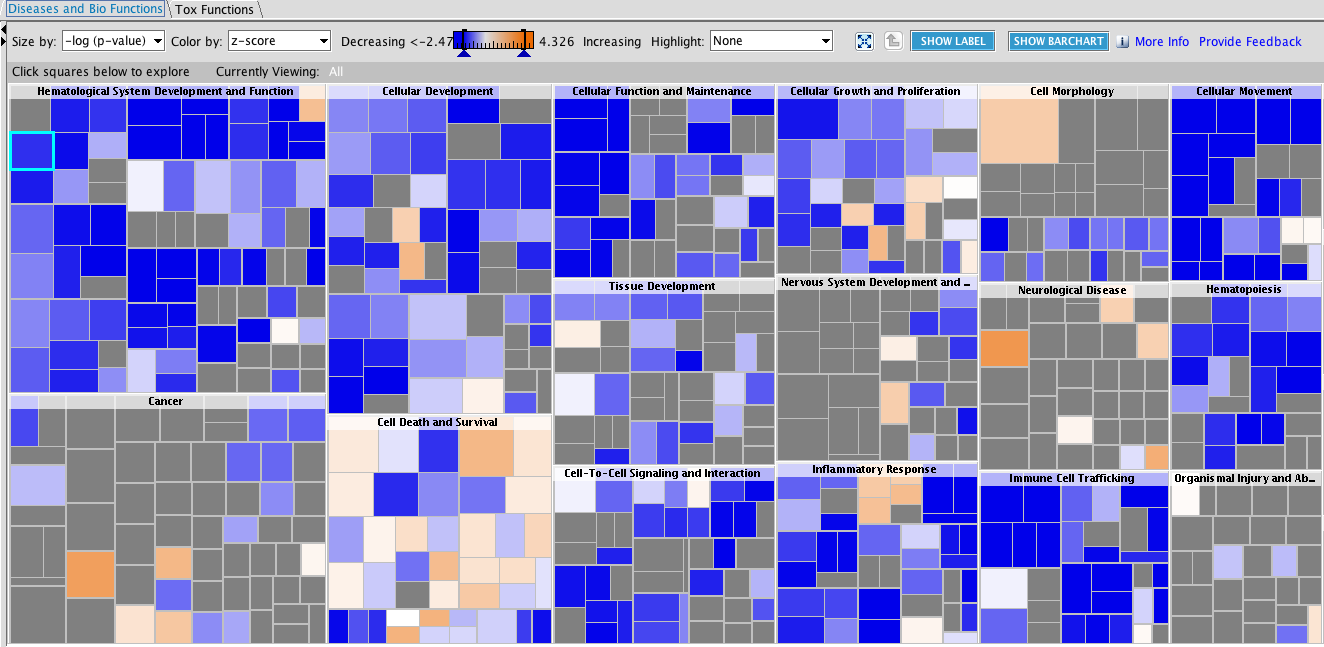


Figure S6: *In vitro* differentially methylated and differentially expressed 238 genes with fold changes are uploaded Ingenuity Pathway Analyses software for functional enrichment. These are the enriched most categories; blue sub-categories are inhibited and orange ones are activated *in vitro*.
