# Supplementary material for: Micro-Environment Causes Reversible Changes in DNA Methylation and mRNA Expression Profiles in Patient-Derived Glioma Stem Cells
Source: PLoS One. 2014 Apr 11;9(4):e94045. doi: 10.1371/journal.pone.0094045 (PMC3984100; doi:10.1371/journal.pone.0094045)
Supplement: Figure S7 — PRMT5 expression for matched PT, in vitro, in vivo and ex vivo samples. Each column represents a matched samples and y-axis is the PRMT5 expression value. (DOCX) [file pone.0094045.s007.docx]

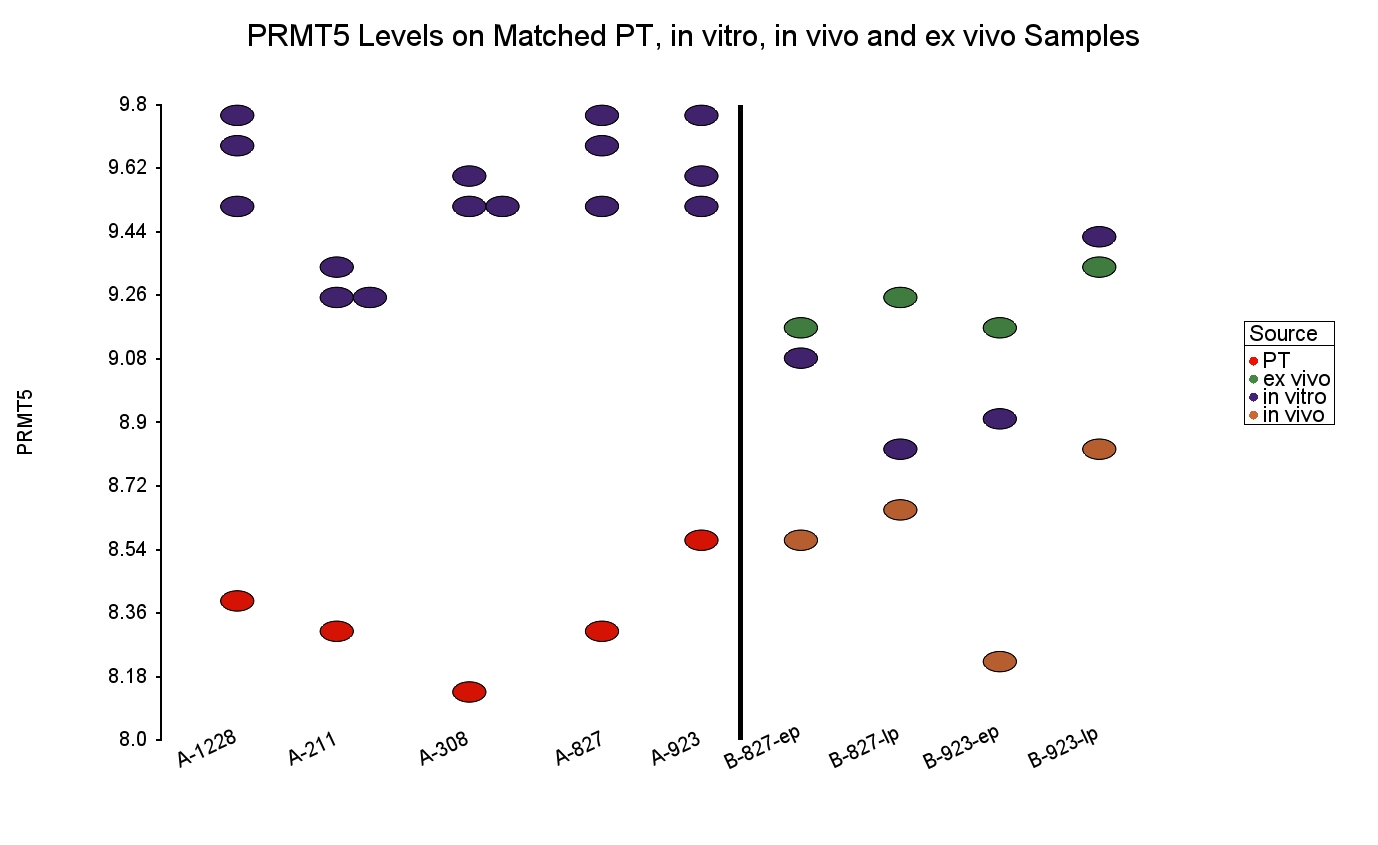


Figure S7: PRMT5 expression for matched PT, *in vitro*, *in vivo* and *ex vivo* samples. Each column represents a matched samples and y-axis is the PRMT5 expression value.
